# Short-term effectiveness of single-dose intranasal spray COVID-19 vaccine against symptomatic SARS-CoV-2 Omicron infection in healthcare workers: a prospective cohort study

**DOI:** 10.1016/j.eclinm.2023.102374

**Published:** 2023-12-13

**Authors:** Hongfei Mi, Qi Chen, Hongyan Lin, Tingjuan He, Ruixin Zhang, Shuhao Ren, Lingling Liu, Jing Wang, Hua Huang, Meixia Wang, Zhinan Guo, Chenghao Su

**Affiliations:** a Zhongshan Hospital, Fudan University (Xiamen Branch), Xiamen, 361015, China; b Xiamen Clinical Research Center for Cancer Therapy, Xiamen, 361015, China; c School of Public Health, Xiamen University, Xiamen, 361102, China; d Xiamen Center for Disease Control and Prevention, Xiamen 361021, China

**Keywords:** Effectiveness, Intranasal spray COVID-19 vaccine, dNS1-RBD, Single-dose regimen, SARS-CoV-2

## Abstract

**Background:**

The pivotal phase 3 efficacy clinical trial has demonstrated that a two-dose regimen of dNS1-RBD (Beijing Wantai Biological Pharmacy Enterprise, Beijing, China) is well-tolerated and provides wide protection against SARS-CoV-2 infection. However, the effectiveness of a single-dose regimen is still unknown. We aimed to estimate the effectiveness of one-dose of dNS1-RBD against symptomatic Omicron infections in real-world conditions.

**Methods:**

This prospective cohort study was conducted during an Omicron outbreak among healthcare workers in Xiamen, China, from December 22, 2022 to January 16, 2023. Participants chose to receive single-dose of dNS1-RBD or remain unvaccinated based on personal preference. Healthcare workers daily validated their SARS-CoV-2 infection status, using either RT-PCR or rapid antigen test. A survey questionnaire was conducted to gather information on acute symptoms from individuals infected with SARS-CoV-2. The primary outcome was the symptomatic SARS-CoV-2 infections after enrollment in the dNS1-RBD recipients or the control group among all participants and by prior COVID-19 vaccination status.

**Findings:**

On December 22, 2022, a total of 1391 eligible participants without a history of prior SARS-CoV-2 infection were enrolled. Among them, 550 received single-dose of dNS1-RBD, while 841 remained unvaccinated. In the total cohort, the range of follow-up time was 1∼26 days. During the study period, a total of 880 symptomatic SARS-CoV-2 infections were identified in the total cohort. The adjusted vaccine effectiveness against symptomatic SARS-CoV-2 infections and the infections requiring medical attention were 19.0% (95% CI: 6.7, 29.7, *P* = 0.004) and 59.4% (95% CI: 25.1, 78.0, *P* = 0.004) in the total cohort, 11.6% (95% CI: −2.4, 23.7, *P* = 0.100) and 55.3% (95% CI: 15.3, 76.4, *P* = 0.014) in the participants with inactivated COVID-19 vaccination history, as well as 87.0% (95% CI: 72.6, 93.9, *P* < 0.001) and 84.2% (95% CI: −41.8, 98.2, *P* = 0.099) in the naïve participants, respectively.

**Interpretation:**

When administered as a booster to individuals with a history of inactivated COVID-19 vaccination, a single-dose of dNS1-RBD provides protection against infections requiring medical attention at least in the short-term after vaccination. The data also showed that a single-dose of dNS1-RBD is protective against symptomatic SARS-CoV-2 infections as a primary immunization for individuals without prior exposure, but due to the limited sample size of naïve participants, further research with a larger sample size is needed to make a solid conclusion.

**Funding:**

10.13039/100010166Xiamen Science and Technology Bureau 2022 General Science and Technology Plan Project and the 10.13039/100000865Bill & Melinda Gates Foundation.

**Research in context**

**Evidence before this study**

We searched PubMed for studies of mucosal COVID-19 vaccines from the inception of the database to November 20, 2023, with the search terms “(COVID-19 OR SARS-CoV-2) AND (intranasal OR nasal OR mucosal OR aerosolized OR inhaled) AND (vaccine) AND (efficacy OR effectiveness OR protect) AND (clinical trial OR cohort study OR database study OR real-world study)”. No language restrictions were applied. A pivotal Phase 3 clinical trial of the dNS1-RBD showed that the vaccine efficacy against symptomatic infections (Omicron symptom index ≥3) was 56.3% (95% CI: −6.3, 82.0) among naïve participants, and 35.1% (95% CI 3.4, 56.4) among participants with a history of COVID-19 vaccination after two-dose of dNS1-RBD vaccination. Another study reported the protection of an aerosolized Ad5-nCoV vaccine in adults with two-dose inactivated COVID-19 vaccination history, and showed that a single dose of aerosolized Ad5-nCoV vaccine provided a relative protection of 35.1% (95% CI: 23.0, 45.2) against COVID-19 diseases when compared to the inactivated COVID-19 vaccine.

**Added value of this study**

To our knowledge, this study is the first to report the effectiveness data of a single-dose regimen of dNS1-RBD (a live-attenuated influenza virus vector-based intranasal SARS-CoV-2 vaccine). We evaluated the effectiveness of single-dose of dNS1-RBD against symptomatic SARS-CoV-2 infections during an outbreak of the Omicron variant. The results indicated that a single-dose of dNS1-RBD provides protection against symptomatic SARS-CoV-2 infections as a primary immunization for individuals without prior infection. When administered as a booster to individuals with a history of inactivated COVID-19 vaccination, the vaccine offers protection against infections requiring medical attention at least in the short-term after vaccination.

**Implications of all the available evidence**

Our results found that, in individuals with a history of inactivated COVID-19 vaccination, a single-dose of dNS1-RBD provides protection against infections requiring medical attention at least in the short-term after vaccination. In addition, the data also showed that a single-dose of dNS1-RBD is protective against symptomatic SARS-CoV-2 infections as a primary immunization for individuals without prior exposure, but due to the limited sample size of naïve participants, further research with a larger sample size is needed to make a solid conclusion. This study provides real-world data regarding the use of a mucosal COVID-19 vaccine in humans, broadening the selection of vaccines to combat the ongoing COVID-19 pandemic.

## Introduction

The coronavirus disease 2019 (COVID-19) epidemic has caused serious damage to public health and the global economy.^1^ Since the emergence of the ancestral strain of severe acute respiratory syndrome coronavirus 2 (SARS-CoV-2) in 2019, the virus evolved rapidly and mutated toward increased viral fitness, enhanced transmission kinetics, and immune evasion.^2^^,^^3^ A wealth of evidence indicated that although intramuscular COVID-19 vaccines initially provided significant protection in reducing viral transmission, hospitalizations, and deaths, the ongoing evolution of SARS-CoV-2 variants, especially Omicron sub-lineages, had led to a rapid decline in the effectiveness of both the primary series and booster dose against symptomatic infections.^4^ Although the World Health Organization (WHO) announced that COVID-19 no longer constitutes a public health emergency of international concern (PHEIC) on May 2023, repeated reinfections and an increased risk of post-acute sequelae are persistent challenges to human health, especially for elders and those with underlying medical conditions.^5^^,^^6^ There is an urgent need for safe and broad-spectrum COVID-19 vaccines with high acceptance to tackle the COVID-19 epidemic.^7^

The protection offered by intramuscular COVID-19 vaccines primarily stems from the neutralizing antibody that targets spike protein.^8^ However, the concentration of antibodies in the respiratory tract are 200–500 times lower than that in the circulatory system, which leads to “anatomical escape” of SARS-CoV-2 in the respiratory tract.^9^^,^^10^ The development of mucosal COVID-19 vaccines has garnered significant attention due to the advantage of inducing much stronger localized immunity in the respiratory tract to provide faster protection at the site of virus entry, in contrast to traditional intramuscularly administered vaccines,^11^, ^12^, ^13^ but there is extremely limited data on their application in humans. The initial findings from the large-scale phase 3 clinical trial of a mucosal COVID-19 vaccine, dNS1-RBD (Pneucolin®) demonstrated that two doses of dNS1-RBD had a favorable safety profile and broad protection against the Omicron variant in the general population without the history of SARS-CoV-2 infection.^14^ dNS1-RBD is a live-attenuated influenza virus vector-based intranasal vaccine that is manufactured with a cold-adapted influenza strain (CA4) without non-structural protein 1 (NS1) as the genetic backbone, into which receptor-binding domain (RBD) genes from ancestral SARS-CoV-2 are inserted by gene reassortment. Data from preclinical studies demonstrated that dNS1-RBD can provide broad and rapid protection following experimental challenges with the prototype, Beta variant, and Omicron variant of SARS-CoV-2, preventing severe disease and reducing viral loads in hamsters.^15^ dNS1-RBD demonstrates broad protective features, a favorable safety profile and high acceptability, indicating its potential as a promising addition to the existing vaccine pools in addressing the ongoing COVID-19 pandemic. On December 2, 2022, dNS1-RBD obtained the Emergency Use Authorization (EUA) in China. On December 14, 2022, the national implementation program of the second booster dose of COVID-19 vaccine was released, in which a series of licensed or EUA COVID-19 vaccines including the dNS1-RBD vaccine were recommended for booster immunization.^16^

Previous studies administered dNS1-RBD in a two-dose regimen. Given the current complex infection context and widespread vaccine hesitancy among the general public, a single-dose regimen may improve the maneuverability of regular booster vaccinations. Nevertheless, there is no available data regarding the use of one-dose regimen of dNS1-RBD in humans to date. Following the implementation of new prevention and control strategies for COVID-19, China experienced a significant surge in infections caused by the Omicron variant, which rapidly peaked in late December 2022 and early January 2023. During the brief outbreak, we conducted this prospective cohort study to estimate the effectiveness of one-dose of dNS1-RBD against symptomatic Omicron infections in real-world conditions.

## Methods

### Study design and participants

This was a prospective cohort study designed to estimate the effectiveness of one-dose of dNS1-RBD against symptomatic SARS-CoV-2 infections among healthcare workers and sub-cohorts with or without COVID-19 vaccination history during the outbreak of the Omicron variant. This study was conducted at the Xiamen Branch, Zhongshan Hospital, Fudan University (ZSXM), which is a public comprehensive tertiary hospital located in Xiamen City, China. Eligible participants were required to meet the following criteria at enrollment: (1) Healthcare workers in ZSXM; (2) Aged 18 years or above; (3) Without prior history of SARS-CoV-2 infection; (4) Had never received any COVID-19 vaccine or have only received inactivated COVID-19 vaccine; (5) Be healthy or have stable underlying medical conditions; (6) Not pregnant or lactating women; (7) Be able to comply with all study procedures.

The study protocol and other study materials related to participants were approved by the Ethical Committee of ZSXM (Approval number: B2022-077). The study was conducted in accordance with the principles of the Declaration of Helsinki, and all participants provided informed consent prior to screening for eligibility.

### Vaccination

dNS1-RBD has been well-characterized previously.^17^ Briefly, this vaccine is a liquid preparation (0.2 mL per vial) and is stored at −15 °C or below. Participants were administered by intranasal spray with a sprayer (0.1 mL per nasal cavity). This vaccine contains 1 × 10⁷ cell culture infective dose 50% of dNS1-RBD per mL.

### Study procedures

On December 22, 2022, healthcare workers underwent screening for eligibility, and after enrollment, based on their personal will, eligible participants were vaccinated with one dose of dNS1-RBD or remained unvaccinated and were included in the vaccine group or control group accordingly. According to the internal SARS-CoV-2 screening program of the ZSXM, oropharyngeal swabs from all of the healthcare workers were collected daily and tested for SARS-CoV-2 infection with the reverse transcription polymerase chain reaction (RT-PCR, Zybio Inc., Chongqing, China) by ZSXM's clinical laboratory, RT-PCR results were reported within 6 hours after sample collection. However, due to the rapidly evolving COVID-19 pandemic, the screening program underwent changes on December 26, 2022, allowing healthcare workers to confirm their SARS-CoV-2 infection status through either RT-PCR in hospital or rapid antigen test (Xiamen Biotime Biotechnology Co., Ltd, Xiamen, China) by themselves (Supplementary Method 1). The follow-up period was expected to encompass the rapid surge of the COVID-19 pandemic. As anticipated, following the subsidence of the COVID-19 outbreak, the screening program had been discontinued since January 17, 2023, after which the systematic collection of daily health status and test results had become significantly challenging, resulting in the end of this study with a relatively brief follow-up period (26 days). The follow-up period for each participant was defined as ending either at the end of the study or upon testing positive for SARS-CoV-2.

In addition, healthcare workers were requested to report their daily health condition and sick leave status on the hospital office automation system every day. A survey questionnaire was conducted to gather information regarding the category, duration, and severity of symptoms, from individuals diagnosed with SARS-CoV-2 infection. Data were retrieved and consolidated from multiple sources, including the hospital office automation system, Xiamen immunization planning and management cloud platform, ZSXM laboratory databases, and survey questionnaires. To ensure data integration, the identity number of each participant was linked as a unique identification code. Authorized personnel from the study team performed the quality control for data collection and integration (Supplementary Method 2).

### Statistical analysis

Continuous variables are reported as the mean with standard deviation (SD) for normally distributed variables, and as the median with interquartile range (IQR) for non-normally distributed variables. Categorical variables are presented as the number with the percentage of participants in each group. The groups were compared using *t*-test for normally distributed continuous variables, Wilcoxon rank sum test for non-normally distributed variables, and *χ*^*2*^ tests for categorical variables. All missing data were not imputed.

The primary outcome of this study was the symptomatic SARS-CoV-2 infections after enrollment in the dNS1-RBD recipients or the control group among the total cohort or sub-cohorts with or without COVID-19 vaccination history. The Cox proportional hazards models were used to evaluate the vaccine effectiveness (VE), with the VE estimated as 1 minus the hazard ratio (1-HR) and 95% confidence interval (95% CI) similarly transformed. Age and other baseline characteristics (*P* < 0.1 in the baseline balance assessment) were included as independent covariates in the model for estimating the adjusted VE. Cumulative incidence curves for the vaccine and control groups were estimated using the Kaplan–Meier method and the 95% CIs for each curve were estimated using the log–log transformation method. The follow-up time, starting from the date of enrollment (December 22, 2022), was used in both Cox proportional hazards models and Kaplan–Meier curves. Participants who failed to undergo virus infection screening nor report their daily health status as requested were considered as lost to follow-up and excluded from the analysis. Furthermore, in the participants who experienced symptomatic SARS-CoV-2 infection, logistic regression model was used to explore the potential impact of dNS1-RBD vaccination on the acute symptoms. Vaccine group and age were forced into the multivariable logistic model, and other baseline characteristics were selected if they were statistically significant with a *P* < 0.1 in the univariate analysis. Wilcoxon rank sum test was used to estimate the difference in the durations of COVID-19 symptoms between two groups. The sample size for this study was determined using the Score (Gart, Nam) method.^18^ In accordance with the 2020 WHO guidelines,^19^ we adhered to the following assumptions: (a) in the total population, a VE of 40.0% against symptomatic SARS-CoV-2 infections with a lower 95% CI of 30.0%, (b) a one-month symptomatic infection rate of 50.0% in the control group, (c) a two-sided α of 0.05, (d) the ratio of vaccine group and control group of 1:2 (according to the results of pre-survey), (e) a screening failure rate of 10%, and (f) a dropout rate of 5%. Accordingly, this study required a sample size of 1379 individuals for enrollment screening.

The latent periods of Omicron infection are usually less than 3 days,^20^ and on the other hand, the vaccine may not reach full effectiveness on the day it is received. A sensitivity analysis was performed by excluding confirmed SARS-CoV-2 infections that occurred within 3 days post vaccination to assess the robustness of the results. Symptomatic SARS-CoV-2 infection was defined as individuals who have tested positive for SARS-CoV-2 through RT-PCR or rapid antigen test, with any of the following symptoms: axillary temperature greater than 37.3 °C, cough, stuffy/runny nose, sore throat, upset stomach, muscle pain, weakness/fatigue, loss of taste or smell, anorexia/nausea/vomiting, headache, diarrhea, chest tightness/shortness of breath, as well as other symptoms diagnosed by the researchers as associated with SARS-CoV-2 infection. Participants assessed the severity of symptoms based on their own health condition, categorizing them into grade 1 (tolerable, no medication needed), grade 2 (requiring medication), and grade 3 or higher (requiring medical attention).

Statistical analyses were conducted using the R programming language 4.2.1 version. All reported tests were 2-sided and a *P* value of less than 0.05 was considered significant.

### Role of the funding source

The funder of the study had no role in study design, data collection, data analysis, data interpretation, or writing of the report.

## Results

### Study participants

**Fig. 1:**
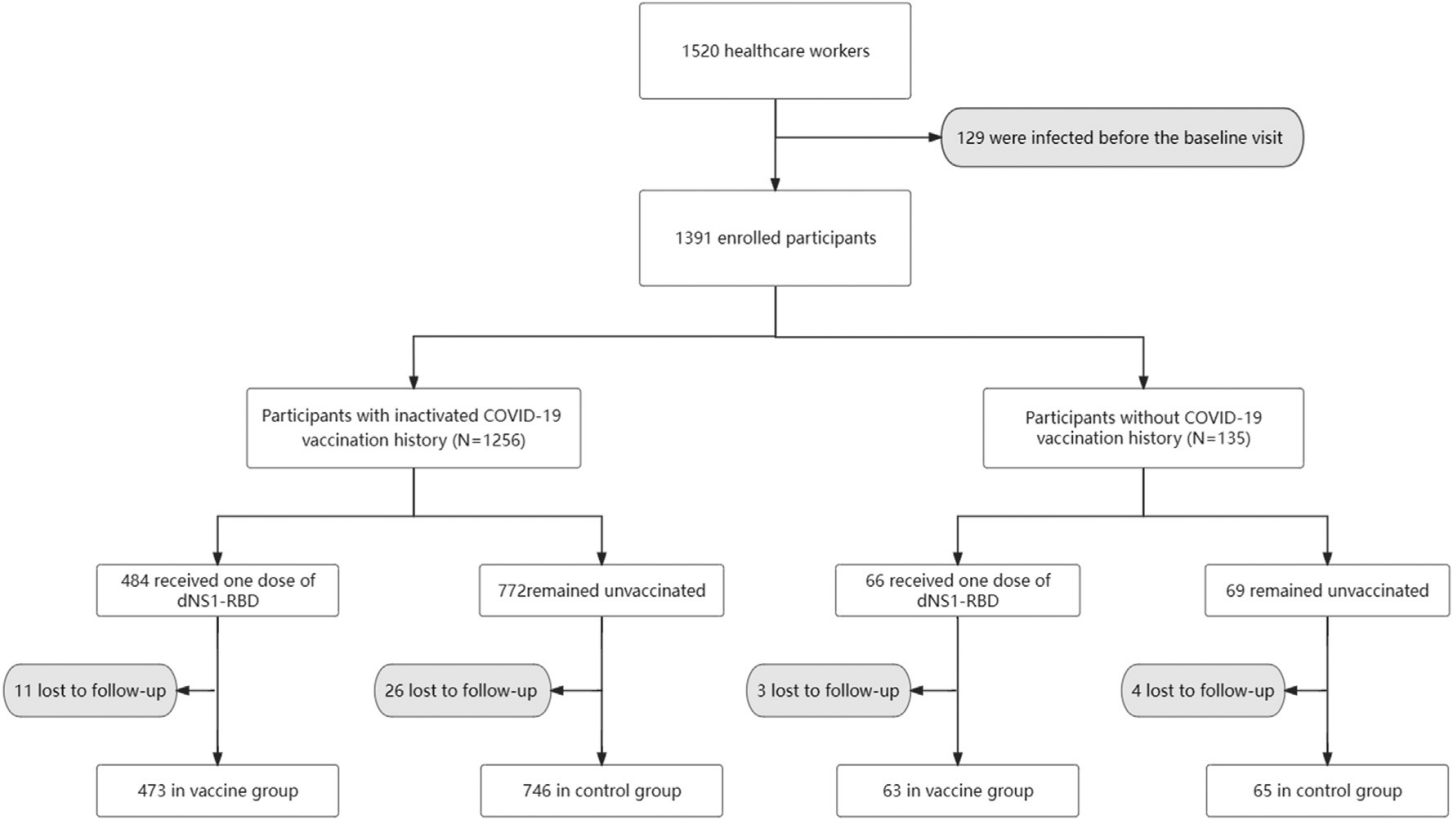
**Study profile**. For the participants with inactivated COVID-19 vaccination history, there were 473 in the vaccine group and the follow-up time was 6418 person-days, as well as 746 in the control group and the follow-up time was 9207 person-days; For the Naïve participants, there were 63 in the vaccine group and the follow-up time was 1182 person-days, as well as 65 in the control group and the follow-up time was 623 person-days. During the study period, a total of 44 participants failed to undergo virus infection screening nor report their daily health status as requested, which we defined as lost to follow-up.

Out of the initial 1520 healthcare workers who underwent eligibility screening, 129 individuals were excluded due to a history of SARS-CoV-2 infection. Finally, 1391 eligible participants were enrolled with 1256 having a history of inactivated COVID-19 vaccination (484 received one dose of dNS1-RBD and 772 remained unvaccinated) and 135 naïve participants who had never received COVID-19 vaccine (66 received one dose of dNS1-RBD and 69 remained unvaccinated). During the study period, a total of 44 participants failed to undergo virus infection screening nor report their daily health status as requested. Therefore, 1347 participants with valid follow-up data were included in the analysis (Fig. 1). The baseline characteristics of the participants are presented in Table 1. Participants in the vaccine group were older compared with those in the control group (37.6 years vs 36.1 years, *P* = 0.015). More than 80% of participants had received three doses of the inactivated COVID-19 vaccine. There was no difference in the constituent ratios of RT-PCR (73.4% vs 72.2%) and rapid antigen test (26.6% vs 27.8%) in the confirmed COVID-19 cases between the groups (*P* = 0.700).

**Table 1:** Baseline characteristics of study participants.

	Total population			Participants with inactivated COVID-19 vaccination history			Naïve participants^a^		
	Vaccine group (N = 536)	Control group (N = 811)	*P* value	Vaccine group (N = 473)	Control group (N = 746)	*P* value	Vaccine group (N = 63)	Control group (N = 65)	*P* value
**Age, mean (SD**)	37.6 (11.3)	36.1 (10.6)	0.015	37.3 (10.9)	35.7 (10.4)	0.013	40.1 (13.8)	40.9 (12.1)	0.734
**Age group, n (%)**									
18–59 years	511 (95.3)	787 (97.0)	0.102	452 (95.6)	725 (97.2)	0.130	59 (93.7)	62 (95.4)	0.666
≥60 years	25 (4.7)	24 (3.0)	–	21 (4.4)	21 (2.8)	–	4 (6.4)	3 (4.6)	–
**Sex, n (%)**									
Male	173 (32.3)	246 (30.3)	0.451	148 (31.3)	218 (29.2)	0.443	25 (39.7)	28 (43.1)	0.697
Female	363 (67.7)	565 (69.7)	–	325 (68.7)	528 (70.8)	–	38 (60.3)	37 (56.9)	–
**Doses of inactivated COVID-19 vaccine previously received, n (%)**									
Unvaccinated	63 (11.8)	65 (8.0)	<0.001	0 (0.0)	0 (0.0)	<0.001	63 (100.0)	65 (100.0)	–
One or two dose	25 (4.7)	94 (11.6)	–	25 (5.3)	94 (12.6)	–	0 (0.0)	0 (0.0)	–
Three doses	448 (83.6)	652 (80.4)	–	448 (94.7)	652 (87.4)	–	0 (0.0)	0 (0.0)	–
**Time since the last dose of inactivated COVID-19 vaccine (Days), median (IQR)**									
	–	–	–	423 (350, 433)	416 (284, 433)	0.010	–	–	–
**Symptomatic SARS-CoV-2 infection, n (%)**									
Total number of cases	323 (60.3)	557 (68.7)	–	304 (64.3)	506 (67.8)	–	19 (30.2)	51 (78.5)	–
Hospitalized or more severe COVID-19 (N)	0	0	–	0	0	–	0	0	–
**Diagnostic tests, n (%,)**^b^									
RT-PCR	237 (73.4)	402 (72.2)	0.700	221 (72.7)	357 (70.6)	0.513	16 (84.2)	45 (88.2)	0.655
Rapid antigen test	86 (26.6)	155 (27.8)	–	83 (27.3)	149 (29.5)	–	3 (15.8)	6 (11.8)	–

N, number of participants; %, proportion of participants; IQR, interquartile range; SD, standard deviation.

a Naïve participants: Participants without COVID-19 vaccination history before enrollment.

b The constituent ratios of diagnostic tests among participants with symptomatic SARS-CoV-2 infection.

### The effectiveness of dNS1-RBD against symptomatic SARS-CoV-2 infections

**Fig. 2:**
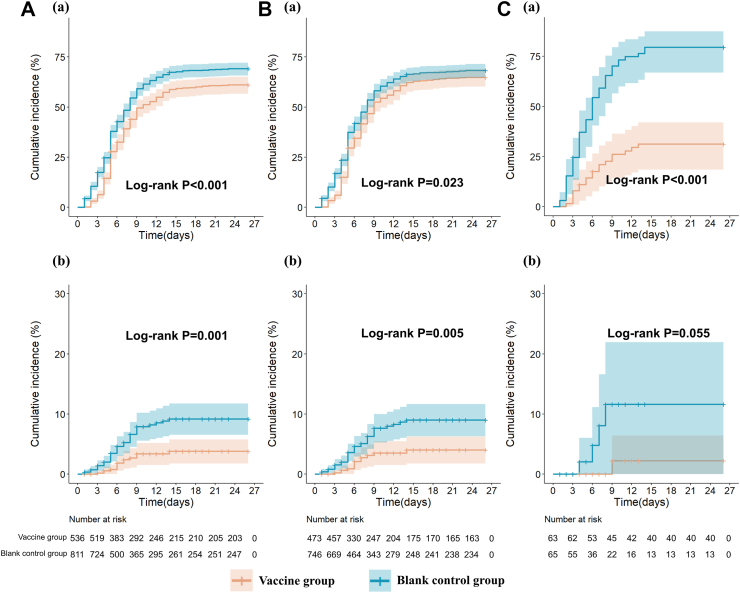
**Cumulative Incidence of SARS-CoV-2 symptomatic infections and those with at least one symptom graded as 3 or higher**. A: Total population; B: Participants with inactivated COVID-19 vaccination history; C: Naïve participants; (a) All the symptomatic infection events; (b) Infection events with at least one symptom ≥ grade 3 (requiring medical attention).

In the total cohort regardless of vaccination history, the range of follow-up time was 1∼26 days. During the observation period, a total of 323 and 557 symptomatic SARS-CoV-2 infections were identified in the vaccine group and control group (incidence rate, 4.3 vs 5.7 per 100 person-days), respectively, with the VE of 23.6% (95% CI: 12.4, 33.4, *P* < 0.001) and the adjusted VE of 19.0% (95% CI: 6.7, 29.7, *P* = 0.004; Table 2). Among the SARS-CoV-2 infected patients, 62 experienced symptoms of grade 3 or higher, of which 14 in the vaccine group and 48 in the control group, with the adjusted VE of 59.4% (95% CI: 25.1, 78.0, *P* = 0.004; Table 2). The cumulative incidence of SARS-CoV-2 symptomatic infections and those with at least one symptom of grade 3 or higher are shown in Fig. 2.

**Table 2:** The effectiveness of dNS1-RBD against symptomatic SARS-CoV-2 infections

	Vaccine group				Control group				VE (95% CI)	*P* value	Adjusted VE^e^ (95% CI)	*P* value
	n	N	Person time^a^	Incidence rate^b^	n	N	Person time^a^	Incidence rate^b^				
**Total population**												
Total cases^c^	323	536	7600	4.3	557	811	9830	5.7	23.6 (12.4, 33.4)	<0.001	19.0 (6.7, 29.7)	0.004
Participants with at least one symptom of severity ≥3^d^	14	536	7600	0.2	48	811	9830	0.5	61.5 (30.2, 78.8)	0.002	59.4 (25.1, 78.0)	0.004
**Participants with inactivated COVID-19 vaccination history**												
Total cases^c^	304	473	6418	4.7	506	746	9207	5.5	14.6 (1.5, 25.9)	0.030	11.6 (−2.4, 23.7)	0.100
Participants with at least one symptom of severity ≥3^d^	13	473	6418	0.2	44	746	9207	0.5	57.9 (21.9, 77.3)	0.006	55.3 (15.3, 76.4)	0.014
**Naïve participants**												
Total cases^c^	19	63	1182	1.6	51	65	623	8.2	74.7 (56.9, 85.1)	<0.001	87.0 (72.6, 93.9)	<0.001
Participants with at least one symptom of severity ≥3^d^	1	63	1182	0.1	4	65	623	0.6	84.6 (−38.8, 98.3)	0.095	84.2 (−41.8, 98.2)	0.099

N, number of participants; n, number of cases.

a The unit of person time was days.

b The unit of incidence rate was per 100 person-days.

c Participants had at least one of the following symptoms: axillary temperature greater than 37.3 °C, cough, stuffy/runny nose, sore throat, upset stomach, muscle pain, weakness/fatigue, loss of taste or smell, anorexia/nausea/vomiting, headache, diarrhea, chest tightness/shortness of breath, as well as other symptoms identified by the researchers as associated with SARS-CoV-2 infection.

d The participants assessed the severity of symptoms as grade 1 (tolerable, no medication needed), grade 2 (requiring medication), and grade 3 or higher (requiring medical attention) based on their own health condition.

e For the total population, age and previous vaccine dose (doses of inactivated COVID-19 vaccine previously received) were included as independent covariates in the model for estimating the adjusted VE; For the participants with inactivated COVID-19 vaccination history, age, previous vaccine dose and vaccine interval (time since the last dose of inactivated COVID -19 vaccine) were included as independent covariates in the model for estimating the adjusted VE; For Naïve participants, only age was included as independent covariates in the model for estimating the adjusted VE.

Among participants with inactivated COVID-19 vaccination history, the adjusted VE against symptomatic infections and the infections with symptom severity graded ≥3 were 11.6% (95% CI: −2.4, 23.7, *P* = 0.100) and 55.3% (95% CI: 15.3, 76.4, *P* = 0.014), respectively (Table 2). In the naïve participants without prior COVID-19 vaccination, the adjusted VE against symptomatic infections and the infections with symptom graded ≥3 were 87.0% (95% CI: 72.6, 93.9, *P* < 0.001) and 84.2% (95% CI: −41.8, 98.2, *P* = 0.099), which were higher compared to those observed in the participants with a history of COVID-19 vaccination. A sensitivity analysis which excluded the SARS-CoV-2 infections that occurred within 3 days post vaccination (Table S1) showed similar results as the main analysis.

### The potential impact of dNS1-RBD vaccination on the acute symptoms

Due to inadequate statistical power and a small sample size of the naïve population, this analysis was only conducted on the participants with a COVID-19 vaccination history. Among the confirmed symptomatic SARS-CoV-2 infection cases in the control group, the five most prevalent symptoms were cough (437/506, 86.4%), fever (408/506, 80.6%), weakness/fatigue (341/506, 67.4%), sore throat (319/506, 63.0%) and stuffy/runny nose (308/506, 60.9%). The dNS1-RBD vaccination was a protective factor against the onset of cough (adjusted OR = 0.65, 95% CI: 0.44, 0.95, *P* = 0.028). It is noteworthy that dNS1-RBD vaccination substantially decreased the incidence of some symptoms with grade 3 or above, which include fever, cough, anorexia, nausea, vomiting, sore throat, headache, and chest tightness/shortness of breath, but most differences did not reach statistical significance due to the limited number of cases within each group (Table S2). Furthermore, our data indicate that dNS1-RBD vaccination appears to reduce the duration of muscle pain (1 day), weakness/fatigue (2 days), and sick leave time (1 day, Table S3).

## Discussion

In this prospective cohort study conducted during the intense surge of Omicron, we examined the effectiveness of a single-dose regimen of dNS1-RBD in health workers who had no previous history of SARS-CoV-2 infection. This is the first report to investigate the effectiveness of a single-dose regimen of dNS1-RBD in preventing symptomatic SARS-CoV-2 infections.

As expected, one-dose of dNS1-RBD provides higher protection against infections requiring medical attention compared to mildly symptomatic infections. Since the majority of healthcare workers in this study were young (the mean age was 37.6 years and 36.1 years in vaccine and control group, respectively), no hospitalized or more severe COVID-19 cases were reported. Among the monitored COVID-19 related symptoms in this study, most of them include cough, stuffy/runny nose, sore throat, upset stomach, muscle pain, weakness/fatigue, loss of taste or smell, anorexia/nausea, headache, chest tightness/shortness of breath, could hardly be graded by an objective standard. According to the WHO Criteria for Grading of COVID-19 Cases,^21^ symptomatic cases that do not require hospitalization were classified as Grade 2 (mild symptoms, not requiring treatment) and Grade 3 (mild symptoms, requiring treatment). Taking into account the healthcare workers' basic medical knowledge and the high workload during the pandemic, the current grading criteria were selected for monitoring all symptoms. However, it is important to note that subjective bias in participants' self-rating of symptoms could not be completely eliminated.

Among the participants with breakthrough SARS-CoV-2 infection, the dNS1-RBD seems to slightly attenuate local respiratory symptoms, such as cough, stuffy/runny nose and sore throat. Additionally, dNS1-RBD vaccination can reduce the durations of partial symptoms. These may be contributed by the intranasal delivery characteristics of dNS1-RBD, which induces innate immunity and trained immunity, while promoting the development of tissue-resident memory T cells in the upper and lower respiratory tract. In animal models, dNS1-RBD also inhibits the inflammatory response by suppressing early phase viral load post SARS-CoV-2 infection and attenuating pro-inflammatory cytokine levels (*Il6, Il1b, and Ifng*), which reduces the immune-induced tissue injury.^22^ Further investigation of the underlying protection mechanism is needed.

Interestingly, we found that the adjusted VEs against symptomatic infections in the participants with a history of inactivated COVID-19 vaccination were lower compared to those observed in the naïve population. To our knowledge, the same trend has been observed in the multicenter, randomized, double-blind, placebo-controlled, phase 3 clinical trial of dNS1-RBD. Specifically, the VE against symptomatic infections (Omicron symptom index ≥3) among participants with a history of COVID-19 vaccination (VE = 35.1%, N = 12,717) was lower than that of naïve population (VE = 56.3%, N = 13,025) after two-dose of dNS1-RBD vaccination.^14^ Further research is needed to investigate the veracity of this trend and elucidate its underlying mechanisms.

During the study period, China experienced an unprecedented surge in infections caused by the Omicron variant. In the control group, a total of 68.7% of participants were infected with SARS-CoV-2 in a short period (26 days). The impact of high pathogen exposure (including frequency and dose) on VE has previously sparked extensive discussion.^23^ A phase 3 clinical trial of RTS, S/AS01 malaria vaccine study revealed a noteworthy trend: higher point estimates of VE were observed in areas with lower transmission rates, while lower point estimates were noted in areas with higher transmission rates (the point estimates of VE from 17.7% to 66.0% in different areas).^24^ A similar pattern has also been observed in the respiratory virus vaccine. A report described the epidemiologic features of the mumps outbreak that occurred among U.S. communities during 2009 and 2010, and showed that intense exposures facilitated mumps virus transmission and overcame vaccine-induced protection.^25^ Notably, a meta-analysis study found that the protection provided by SARS-CoV-2 infection in healthcare workers (high work-related exposure risk) was relatively lower compared to the general population.^26^ Therefore, it's reasonable to hypothesize that the effectiveness observed would not be overestimated. Further study in the lower transmission conditions is necessary.

The phase 3 clinical trial of dNS1-RBD was conducted in the Philippines, South Africa, Colombia and Vietnam since December 16, 2020, during which the top three circulating SARS-CoV-2 sub-lineages successfully sequenced in this trial were BA.4.1 (31.2%), BA.2 (15.6%) and BA.2.3 (13.8%). According to the data from the Global Initiative on Sharing All Influenza Data (GISAID),^27^ all of the circulating strains were sub-lineages of Omicron in Xiamen City during the study period. Specifically, the predominant sub-lineages were BA.5.2.48^∗^ (68.9%, included BA.5.2.48, DY.1, DY.1.1, DY.2, DY.3 and DY.4) and BF.7.14^∗^ (26.7%, included BF.7.14, BF.7.14.1, BF.7.14.3, BF.7.14.5, BF.7.14.6), which exhibited approximately 100 mutations compared with the prototype and were demonstrated about 20-fold increase in fitness compared to the prototype.^28^, ^29^, ^30^ dNS1-RBD showed a modest protection against the emerging SARS-CoV-2 sub-lineages in Xiamen City, further confirming the broad-spectrum characteristic of this intranasal spray vaccine.

Vaccination plays an indispensable role in the control of the COVID-19 pandemic and has saved millions of lives.^31^ However, due to vaccine hesitancy or contraindications to intramuscular vaccines, there remains a substantial proportion of people especially the vulnerable and elder population who are reluctant to take them, even COVID-19 vaccines are free and easily accessible.^32^ Numerous studies indicated that fear of injection was also one of the main determinants of vaccine hesitancy.^33^ dNS1-RBD provides a new option for those individuals, with the advantages of being needle-free and non-invasive. A recent survey study on the acceptability of dNS1-RBD included 10,452 participants, among whom 92.6% reported no discomfort during the inoculation, and 99.8% thought the vaccination process went well. 58.8% of the participants preferred the intranasal spray, 8.4% preferred the intramuscular injection, and 32.9% had no preferences.^34^ The previous clinical trials demonstrated that dNS1-RBD was well tolerated in the elderly population aged ≥60 years and individuals with underlying medical conditions, including hypertension, diabetes, heart disease, asthma, and so on.^14^ dNS1-RBD has the potential to significantly reduce vaccine hesitancy and increase immunization coverage, particularly among vulnerable populations with the requirement of regular boosting.

The strengths of this study include the availability of daily reported systemic SARS-CoV-2 screening data and clinical symptoms among healthcare workers, allowing the accurate assessment of the VE against symptomatic infections with different severity and the potential impact of dNS1-RBD vaccination on the acute symptoms in real-world conditions, especially during the first intense wave of the COVID-19 pandemic in China. Due to the “dynamic Zero-COVID” policy, SARS-CoV-2 infection rate remained extremely low before the initiation of this study. In Xiamen, healthcare workers underwent regular nucleic acid testing at a minimum frequency of once per week during the period of “dynamic Zero-COVID” policy. Starting from November 28, 2022, the testing frequency was increased to once a day in ZSXM. As a result, participants with unrecognized asymptomatic SARS-CoV-2 infection history were uncommon. Consequently, most if not all of the enrolled health workers had no naturally acquired immunity against SARS-CoV-2 prior to the study, which minimized the possible effect of immunity induced by undetected natural infections on the study outcomes. Importantly, amidst the ongoing COVID-19 pandemic in China, there has been a growing number of SARS-CoV-2 infections. Further investigation is required to gauge the level of protection offered by the single dose regimen in individuals with hybrid immunity resulting from SARS-CoV-2 infection and vaccination.

This study has several limitations. First, this study is not a randomized blinded study. Although the Cox proportional hazards models were adjusted for potential confounders, the differences in factors between vaccine and control groups, including age and baseline immunization situation, can still confound the results. For example, the individuals in the vaccine group may be more health-conscious than those who choose not to be vaccinated, regardless of their baseline immunization history. In addition, increased age has been reported to be a risk factor for the severity of COVID-19. After SARS-CoV-2 infection, older individuals presented a higher likelihood of exhibiting symptoms and being identified as symptomatic COVID-19 cases.^35^ This unequal probability between the two groups might slightly underestimate the effectiveness of dNS1-RBD. Second, this estimate is based on a relatively brief follow-up period (26 days). Third, due to the rapidly evolving COVID-19 pandemic, healthcare workers were allowed to confirm their SARS-CoV-2 infection status through either RT-PCR or rapid antigen test by themselves after December 26, 2022. However, the proportions of RT-PCR and rapid antigen test were similar between two groups, which was well-balanced. Fourth, the inherent characteristics of a single-center, research participant and small sample size cohort study (especially naïve population) might limit the generalizability of the findings.

In conclusion, when administered as a booster to individuals with a history of inactivated COVID-19 vaccination, a single-dose of dNS1-RBD provides protection against infections requiring medical attention at least in the short-term after vaccination. The data also showed that a single-dose of dNS1-RBD is protective against symptomatic SARS-CoV-2 infections as a primary immunization for individuals without prior exposure, but due to the limited sample size of naïve participants, further research with a larger sample size is needed to make a solid conclusion.

## Contributors

HFM, HYL, TJH, MXW, ZNG, and CHS designed the study. HFM, TJH, LLL, JW, HH, and CHS were involved in the running of the study and in recruiting study participants. HFM, QC, TJH, MXW, ZNG, and CHS verified the data. All authors had access to the raw data. QC, RXZ, SHR and TJH performed statistical analyses. HFM and QC drafted the manuscript which was reviewed, revised, and approved by all authors. All authors were responsible for the final decision to submit the manuscript and vouched for the accuracy and completeness of the data, for adherence of the study to the protocol.

## Data sharing statement

The data in this Article will be available after publication. Researchers who provide a scientifically sound proposal will be allowed to access the de-identified individual participant data. Proposals should be sent to the corresponding author Chenghao Su (su.chenghao@zsxmhospital.com). Proposals will be reviewed and approved by the study team.

## Declaration of interests

All authors declare no competing interests.
