# Supplementary material for: Short-term effectiveness of single-dose intranasal spray COVID-19 vaccine against symptomatic SARS-CoV-2 Omicron infection in healthcare workers: a prospective cohort study
Source: eClinicalMedicine. 2023 Dec 13;67:102374. doi: 10.1016/j.eclinm.2023.102374 (PMC10758709; doi:10.1016/j.eclinm.2023.102374)
Supplement: Supplementary file [file mmc1.docx]

**Supplementary File**

**Short-term effectiveness of single-dose intranasal spray COVID-19 vaccine against symptomatic SARS-CoV-2 Omicron infection in healthcare workers: a prospective cohort study**

**Supplementary Appendix**

**[Table S1. The effectiveness of dNS1-RBD against symptomatic SARS-CoV-2 infections (cases occurring within 3 days after enrollment were excluded)](#_Toc151476032)** [3](#_Toc151476032)

**[Table S2. The impact of dNS1-RBD vaccination on the onset of COVID-19 symptoms](#_Toc151476033)** [5](#_Toc151476033)

**[Table S3. The duration of COVID-19 symptoms in dNS1-RBD recipients and the control participants](#_Toc151476034)** [8](#_Toc151476034)

**[Method S1. Detailed information about the RT-PCR and rapid antigen test used in the study](#_Toc151476035)** [9](#_Toc151476035)

**[Method S2. Quality control procedures of data collection and integration](#_Toc151476036)** [10](#_Toc151476036)


**Table S1. The effectiveness of dNS1-RBD against symptomatic SARS-CoV-2 infections (cases occurring within 3 days after enrollment were excluded)**

|  | Vaccine group | | | |  | Control group | | | | VE (95%CI) | *P* value | Adjusted VE^@^ (95%CI) | *P* value |
| --- | --- | --- | --- | --- | --- | --- | --- | --- | --- | --- | --- | --- | --- |
|  | n | N | Person time* | Incidence rate^#^ |  | n | N | Person time* | Incidence rate^#^ |  |  |  |  |
| **Total population** | | | | | | | | | | | | | |
| Total^&^ | 289 | 502 | 6010 | 4.8 |  | 415 | 665 | 7521 | 5.5 | 10.2 (-4.3, 22.7) | 0.160 | 2.5 (-13.8, 16.6) | 0.745 |
| Participants with at least one symptom of severity ≥ 3^ | 13 | 502 | 6010 | 0.2 |  | 37 | 665 | 7521 | 0.5 | 54.6 (14.5, 75.8) | 0.014 | 56.1 (15.7, 77.1) | 0.013 |
| **Participants with inactivated COVID-19 vaccination history** | | | | |  |  |  |  |  |  |  |  |  |
| Total^&^ | 275 | 444 | 5016 | 5.5 |  | 380 | 617 | 7081 | 5.4 | -1.5 (-18.6, 13.0) | 0.846 | -7.1 (-25.8, 8.7) | 0.399 |
| Participants with at least one symptom of severity ≥ 3^ | 12 | 444 | 5016 | 0.2 |  | 33 | 617 | 7081 | 0.5 | 49.0 (1.2, 73.6) | 0.046 | 50.9 (2.4, 75.3) | 0.042 |
| **Naïve participants** |  |  |  |  |  |  |  |  |  |  |  |  |  |
| Total^&^ | 14 | 58 | 994 | 1.4 |  | 35 | 48 | 440 | 8.0 | 76.1 (55.4, 87.2) | <0.001 | 76.7 (56.4, 87.6) | <0.001 |
| Participants with at least one symptom of severity ≥ 3^ | 1 | 58 | 994 | 0.1 |  | 4 | 48 | 440 | 0.9 | 84.6 (-38.8, 98.3) | 0.095 | 84.2 (-41.8, 98.2) | 0.099 |

N, number of participants; n, number of cases;

* The unit of person time was days; # The unit of incidence rate was per 100 person-days;

&Participants had at least one of the following symptoms: axillary temperature greater than 37.3℃, cough, stuffy/runny nose, sore throat, upset stomach, muscle pain, weakness/fatigue, loss of taste or smell, anorexia/nausea/vomiting, headache, diarrhea, chest tightness/shortness of breath, as well as other symptoms identified by the researchers as associated with SARS-CoV-2 infection

^The participants assessed the severity of symptoms as grade 1 (tolerable, no medication needed), grade 2 (requiring medication), and grade 3 or higher (requiring medical attention) based on their own health condition.

@ For the total population, age and previous vaccine dose (doses of inactivated COVID-19 vaccine previously received) were included as independent covariates in the model for estimating the adjusted VE; For the participants with inactivated COVID-19 vaccination history, age, previous vaccine dose and vaccine interval (time since the last dose of inactivated COVID -19 vaccine) were included as independent covariates in the model for estimating the adjusted VE; For Naïve participants, only age was included as independent covariates in the model for estimating the adjusted VE.

**Table S2.** **The impact of dNS1-RBD vaccination on the onset of COVID-19 symptoms**

|  | Vaccine group (N=304) | Control group (N=506) | OR (95% CI) | *P* value | Adjusted OR^*^ (95% CI) | *P* value |
| --- | --- | --- | --- | --- | --- | --- |
| Fever |  |  |  |  |  |  |
| Total | 244 (80.3) | 408 (80.6) | 0.98 (0.68, 1.40) | 0.898 | 1.07 (0.72, 1.59) | 0.744 |
| Grade 3 or higher | 1 (0.3) | 16 (3.2) | 0.10 (0.01, 0.77) | 0.027 | 0.11 (0.02, 0.80) | 0.029 |
| Cough |  |  |  |  |  |  |
| Total | 245 (80.6) | 437 (86.4) | 0.66 (0.45, 0.96) | **0.030** | 0.65 (0.44, 0.95) | **0.028** |
| Grade 3 or higher | 6 (2.0) | 25 (4.9) | 0.39 (0.16, 0.96) | **0.040** | 0.39 (0.16, 0.96) | **0.040** |
| Stuffy/runny nose |  |  |  |  |  |  |
| Total | 171 (56.3) | 308 (60.9) | 0.83 (0.62, 1.10) | 0.196 | 0.90 (0.66, 1.22) | 0.483 |
| Grade 3 or higher | 5 (1.6) | 6 (1.2) | 1.39 (0.42, 4.61) | 0.586 | 1.46 (0.42, 5.11) | 0.557 |
| Sore throat |  |  |  |  |  |  |
| Total | 174 (57.2) | 319 (63.0) | 0.79 (0.59, 1.05) | 0.101 | 0.83 (0.61, 1.12) | 0.223 |
| Grade 3 or higher | 4 (1.3) | 13 (2.6) | 0.51 (0.16, 1.57) | 0.237 | 0.56 (0.17, 1.82) | 0.337 |
| Upset stomach |  |  |  |  |  |  |
| Total | 65 (21.4) | 93 (18.4) | 1.21 (0.85, 1.72) | 0.297 | 1.27 (0.88, 1.84) | 0.208 |
| Grade 3 or higher | 2 (0.7) | 3 (0.6) | 1.11 (0.18, 6.68) | 0.909 | 1.01 (0.16, 6.26) | 0.993 |
| Muscle pain |  |  |  |  |  |  |
| Total | 178 (58.6) | 300 (59.3) | 0.97 (0.73, 1.30) | 0.837 | 1.03 (0.76, 1.39) | 0.866 |
| Grade 3 or higher | 2 (0.7) | 5 (1.0) | 0.66 (0.13, 3.44) | 0.625 | 0.70 (0.13, 3.90) | 0.686 |
| Weakness /fatigue |  |  |  |  |  |  |
| Total | 200 (65.8) | 341 (67.4) | 0.93 (0.69, 1.26) | 0.639 | 0.99 (0.72, 1.35) | 0.926 |
| Grade 3 or higher | 3 (1.0) | 6 (1.2) | 0.10 (0.01, 0.77) | 0.027 | 0.95 (0.22, 4.23) | 0.950 |
| Loss of taste or smell^#^ |  |  |  |  |  |  |
| Total | 82 (27.0) | 132 (26.1) | 1.05 (0.76, 1.44) | 0.781 | 1.08 (0.77, 1.51) | 0.657 |
| Grade 3 or higher | 1 (0.3) | 1 (0.2) | 1.67 (0.10, 26.74) | 0.718 | 1.74 (0.10, 30.56) | 0.704 |
| Anorexia/nausea/vomiting^#^ |  |  |  |  |  |  |
| Total | 65 (21.4) | 101 (20.0) | 1.04 (0.73, 1.48) | 0.844 | 1.15 (0.80, 1.65) | 0.448 |
| Grade 3 or higher | 0 (0.0) | 3 (0.6) | NA | **<0.001** | NA | **<0.001** |
| Headache^^^ |  |  |  |  |  |  |
| Total | 135 (44.4) | 233 (46.1) | 0.94 (0.70, 1.25) | 0.650 | 0.99 (0.73, 1.34) | 0.947 |
| Grade 3 or higher | 1 (0.3) | 8 (1.6) | 0.21 (0.03, 1.65) | 0.137 | 0.21 (0.03, 1.77) | 0.151 |
| Diarrhea |  |  |  |  |  |  |
| Total | 52 (17.1) | 71 (14.0) | 1.26 (0.86, 1.87) | 0.239 | 1.25 (0.83, 1.87) | 0.284 |
| Grade 3 or higher | 1 (0.3) | 1 (0.2) | 1.67 (0.10, 26.74) | 0.718 | 1.51 (0.09, 24.81) | 0.774 |
| Chest tightness/Shortness of breath |  |  |  |  |  |  |
| Total | 26 (8.6) | 42 (8.3) | 1.03 (0.62, 1.72) | 0.900 | 0.91 (0.54, 1.53) | 0.721 |
| Grade 3 or higher | 0 (0.0) | 7 (1.4) | NA | **<0.001** | NA | **<0.001** |
| Other |  |  |  |  |  |  |
| Total | 26 (8.6) | 46 (9.1) | 0.94 (0.57, 1.55) | 0.794 | 0.97 (0.59, 1.61) | 0.913 |
| Grade 3 or higher | 4 (1.3) | 6 (1.2) | 1.11 (0.31, 3.97) | 0.871 | 1.19 (0.3, 4.63) | 0.806 |

^*^ Vaccine group and age were forced into the multivariable logistic model.

^#^In the analysis regarding loss of taste or smell and anorexia/nausea/vomiting, a baseline characteristic (time since the last dose of inactivated COVID -19 vaccine) was statistically significant with a P<0.1 in the univariate analysis and was selected into the multivariable logistic model.

^^^In the analysis regarding headache, a baseline characteristic (doses of inactivated COVID-19 vaccine previously received) was statistically significant with a P<0.1 in the univariate analysis and was selected into the multivariable logistic model.

**Table S3. The duration of COVID-19 symptoms in dNS1-RBD recipients and the control participants**

| Time, Median (IQR) | Vaccine group | Control group | *P* value |
| --- | --- | --- | --- |
| Sick leave time | 5 (2,7) | 6 (3,8) | **<0.001** |
| Fever | 2 (1,3) | 2 (2,3) | 0.209 |
| Cough | 8 (6,12) | 9 (5,14) | 0.58 |
| Stuffy/runny nose | 4 (3,7) | 5 (3,8) | 0.178 |
| Sore throat | 4 (3,7) | 4 (3,7) | 0.521 |
| Upset stomach | 3 (2,5.25) | 3 (2,6) | 0.224 |
| Muscle pain | 3 (2,5) | 4 (2,6) | **0.045** |
| Weakness /fatigue | 4 (2,7) | 6 (4,9) | **0.002** |
| Loss of taste or smell | 7 (4,11) | 6 (5,10) | 0.927 |
| Anorexia/nausea/vomiting | 5 (3,7.25) | 6 (3,9) | 0.241 |
| Headache | 3 (2,6) | 4 (2,6) | 0.343 |
| Diarrhea | 3 (1,5) | 2 (1,5) | 0.753 |
| Chest tightness/Shortness of breath | 5 (2,12.5) | 4 (3,7) | 0.629 |
| Other | 2 (1.5,3.5) | 4 (2,6) | 0.143 |

*Wilcoxon rank sum test was used to estimate the difference in the durations of COVID-19 symptoms between two groups. IQR, interquartile range;

**Method S1. Detailed information about the RT-PCR and rapid antigen test used in the study**

1. RT-PCR (Zybio Inc., Chongqing, China): The registration number is 20213400228 in China. This is a SARS-CoV-2 Nucleic Acid Detection Kit (PCR-Fluorescent Probe Method), which was included in the COVID-19 In Vitro Diagnostic Medical Device and Test Methods Database of the European Commission. The gene targets of this kit were ORF1ab gene and N gene. According to the data shown on the European Commission web (https://covid-19-diagnostics.jrc.ec.europa.eu/devices/detail/2204, updated on August 24, 2022), the clinical sensitivity of this kits was 98.8% and specificity was 100.0%.
2. Rapid antigen test (Xiamen Biotime Biotechnology Co., Ltd, Xiamen, China): The registration number is 20223400682 in China. The SARS-CoV-2 antigen rapid qualitative test is a colloidal gold immunochromatography intended for the qualitative detection of nucleocapsid antigen (N protein) from SARS-CoV-2 in human nasal swabs and throat swabs from individuals. The kits used in our study was approved by Chinese National Medical Products Administration (NMPA) on May 20, 2022. According to the information from the official website of Xiamen Biotime Biotechnology Co., Ltd, the relative clinical sensitivity of this kits was 96.0% and specificity was 100.0%. (https://www.xiamenbiotime.com/sars-cov-2-antigen-rapid-qualitative-test_p72.html).

**Method S2. Quality control procedures of data collection and integration**

-Firstly, the authorized personnel checked the correctness of the identity number.

-Secondly, the authorized personnel integrated the database through matching the identity number of participants.

-Thirdly, the authorized personnel verified the completeness of essential information. For example, complete information regarding whether or not the COVID-19 vaccine had been administered, the date of first vaccination, the type of first vaccination should not be lacking.

-Fourth, the authorized personnel standardized the data by, for example, unifying date formats.

-Fifth, the authorized personnel verified the data with its sources to ensure accuracy and consistency. For example, the consistency between severity of symptoms in merged Databases and survey questionnaires.

-Sixth, the authorized personnel conducted logical checks on key variables. For instance, they assessed if the date of the second vaccination followed the date of the first vaccination.
